# Concealment of juvenile bull trout in response to temperature, light, and substrate: Implications for detection

**DOI:** 10.1371/journal.pone.0237716

**Published:** 2020-09-04

**Authors:** Russell F. Thurow, James T. Peterson, Gwynne L. Chandler, Christine M. Moffitt, Theodore C. Bjornn

**Affiliations:** 1 U.S. Department of Agriculture-Forest Service, Rocky Mountain Research Station, Salmon, Idaho, United States of America; 2 U.S. Geological Survey, Oregon Cooperative Fish and Wildlife Research Unit, Oregon State University, Corvallis, Oregon, United States of America; 3 Department of Fish and Wildlife Sciences, University of Idaho, Moscow, Idaho, United States of America; Pacific Northwest National Laboratory, UNITED STATES

## Abstract

Bull trout (*Salvelinus confluentus)* are challenging to detect as a result of the species cryptic behavior and coloration, relatively low densities in complex habitats, and affinity for cold, high clarity, low conductivity waters. Bull trout are also closely associated with the stream bed, frequently conceal in substrate, and this concealment behavior is poorly understood. Consequently, population assessments are problematic and biologists and managers often lack quantitative information to accurately describe bull trout distributions, estimate abundance, and assess status and trends; particularly for stream-dwelling populations. During controlled laboratory trials, we recorded concealment, resting, and swimming behavior of juvenile wild bull trout in response to: (1) constant and fluctuating water temperature, (2) presence or absence of light, and (3) substrate size. Light level had the strongest influence on wild fish concealment and more fish concealed as light levels increased from darkness to daylight. Wild fish were 14.5 times less likely to conceal in constant darkness and 4.1 times more likely to conceal in 12 h light x 12 h darkness compared to constant light. Wild fish were 6.2 times less likely to conceal in small (26–51 mm) substrate compared to larger (52–102 mm) substrate. As water temperature increased, fewer wild fish concealed. Knowledge of wild bull trout concealment will improve field sampling protocols and increase detection efficiencies. These data also enhance knowledge of bull trout niche requirements which illuminates ecological differences among species and informs conservation and restoration efforts.

## Introduction

During the last three decades, increasing interest in bull trout has expanded research and published literature, and substantially improved knowledge of the species’ life history, ecology, and genetics [e.g., 1–3]. Despite this trend, biologists and managers often lack quantitative information to accurately describe bull trout distributions, estimate abundance, and assess status and trends; particularly for stream-dwelling populations. Population assessments are challenging because very specific habitat requirements and population characteristics make bull trout difficult to sample. Bull trout have an affinity for cold water (<16°C) and many populations reside in streams with low conductivities (<100 μS/cm) and clear water [4–7]. Coloration and cryptic behavior make them difficult to detect and bull trout frequent areas with complex instream cover and coarse substrate [8–10]. Juvenile bull trout are closely associated with the streambed and conceal within substrate [10–13]. Bull trout also tend to be found in densities as low as 0.2 fish per 100m and populations are often clustered in specific areas of suitable habitat [14–17].

The U.S. Fish and Wildlife Service final Bull Trout Recovery Plan [18] concluded bull trout warranted “threatened” species status and outlined conservation actions needed for recovery. The plan also reported the results of a 5-year status review [19] that evaluated the status of 121 bull trout core areas. Population trends in 54.6% (n = 66) of core areas were reported as unknown, 23 were declining (ranging from slight to severe), 18 were stable, and 14 were increasing. Other surveys have confirmed large information gaps in the understanding of bull trout population dynamics, monitoring and evaluation, and community interactions [20]. The authors concluded that obtaining information related to the status, trends, and gaps in current research can be extremely difficult for cryptic species such as bull trout that also exhibit broad spatial patterns. In Alberta, quantitative information to assess abundance and distribution of bull trout was “very limited” and long-term population trend data were “rare” [21]. This lack of adequate population status and trend information may compromise efforts to conserve and restore bull trout populations.

In response to the need for status and trend information, biologists summarized then-existing knowledge of the distribution and status of bull trout across 4,462 subwatersheds in the interior Columbia River basin in Oregon, Washington, Idaho, Montana, and Nevada and in the Klamath River basin in Oregon [22]. Within unsampled areas, the authors used classification trees and patterns of association between known distributions and landscape characteristics to predict bull trout distribution. Subsequently, biologists and managers have continued to utilize a variety of field sampling techniques to more accurately describe bull trout status and trends [e.g., 23, 24]. Others [25] have applied broad-scale temperature data and other environmental covariates to improve predictions of probable bull trout distribution across the Columbia River Basin. Concurrently, environmental DNA (eDNA) sampling has emerged as a promising method for validating bull trout presence and for potentially confirming previously unknown populations [26, 27].

Although eDNA sampling holds promise for accurately estimating bull trout distributions, it is currently unsuitable for obtaining more detailed population information. Two of the most common field methods for obtaining population abundance, size structure, status, or trend data, particularly in wadeable streams [28], include electrofishing and underwater observation by snorkeling [29]. The reliability of both methods is influenced by their ability to capture (i.e., electrofishing) or detect (i.e., snorkeling) bull trout (henceforth, detection efficiency). Detection of bull trout poses problems; they are cryptic in nature and the species concealment behavior may create biases in both inventory methods. Failure to account for differences in detection efficiency introduces a systematic error or bias into data that can significantly affect abundance estimates and models [30]. Most electrofishing removal estimates of bull trout are biased and these biases are related to stream characteristics, fish behavior, and fish size [31]. Mark-recapture approaches [32] require fish handling which may affect fish physiologically and behaviorally [33], thereby altering capture vulnerability and biasing capture efficiency estimates. Similarly, the use of raw day or night bull trout snorkel counts, unadjusted for biases, will result in erroneous conclusions [34]. To avoid systematic errors, biologists suggested adjusting inventory data via empirical data or by modeling the efficiency of selected sampling methods [31, 34].

Population inventories via snorkeling or electrofishing might be improved by adopting empirically-based sampling protocols that account for bull trout concealment behavior and enhance detection. For example, bull trout were detected at significantly lower rates as water temperatures declined [34]. Increased concealment of salmonids into substrate is well documented as water temperatures decline [10–13, 35–37]. Concealment causes fish to be unobservable or more difficult to capture or detect. In some cases, the relationship between detection and environmental and biological factors may be relatively direct. For example, as water temperatures declined below a threshold, fish abandoned feeding positions and concealed to conserve energy [38]. In other cases, concealment may be more complex as multiple factors interact, such as when salmonids become photonegative and conceal during the day [6, 39] while emerging at night [10, 13]. Concealment may also be modified by other factors such as cover availability, predators, prey abundance, and food abundance. Use of substrate for concealment by rainbow trout (*Oncorhynchus mykiss*) was dependent on the availability of suitable-sized substrate [40]. Migrating adult bull trout caused juvenile bull trout to move into shallow water and stream margins, presumably to avoid cannibalism [41]. Although emergence from cover to feed at night may reduce trout predation risk and concentrate feeding during periods of largest drift abundance [42], winter nocturnal behavior of brown trout (*Salmon trutta*) was not primarily determined by food availability [43]. Consequently, several factors may interact to influence concealment rates of bull trout and the species subsequent detection.

A growing body of literature has documented daytime concealment of juvenile bull trout at low water temperatures [10, 12, 13, 44–47]. Concealment causes fish to be unobservable or more difficult to capture or detect. Diel differences in behavior and habitat use have important implications for fisheries surveys, since they may influence the effectiveness of techniques used to determine the distribution and abundance of bull trout [21]. Consequently, a better understanding of the factors that influence concealment behavior and detection of bull trout will assist development of more effective sampling protocols and improve understanding of bull trout life history and ecology. Our objective was to investigate factors that influence the concealment behavior of wild, juvenile bull trout. We conducted trials in laboratory aquaria and observed the behavior of bull trout in response to three variables: (1) constant and fluctuating water temperatures, (2) presence or absence of light, and (3) various substrate sizes. By modifying these variables in a controlled, laboratory setting, we tested the influence of each variable both individually and interactively. Knowledge of bull trout concealment behavior may be applied to improve field sampling protocols and methods to increase detection efficiency. In addition to improving field sampling protocols, increasing knowledge of bull trout concealment behavior also enhances our understanding of bull trout niche requirements. Knowledge of niche requirements is important for understanding a species ecology [48], illuminates the ecological differences that allow species to coexist along ecological gradients, and directly informs efforts to conserve and restore critical habitats.

## Methods

Trials were conducted in sixteen, 100 L glass aquaria in the University of Idaho (UI) Fisheries wet laboratory. Aquaria were 60 cm long x 45 cm wide x 45 cm tall. Pathogen-free dechlorinated water was supplied to each aquarium as a flow-through system at 4–5 L/min. Water was chilled in a trough, delivered to aquaria via a manifold system, drained back to the trough, chilled, and recirculated. Supplemental compressed air was provided with an air stone to keep oxygen near saturation. We lined three sides of each aquarium with opaque black plastic to provide visual isolation between fish in adjacent aquaria.

### Fish source and care

This study was carried out in 1991, in strict accordance with the *Guidelines for the Use of Fishes in Field Research* (ASIH/AFS/AIFRB 1987, 1988) and the *Principles for the utilization and care of vertebrate animals used in testing*, *research*, *and training* (United States Interagency Research Animal Committee, Federal Register. 1985; 50(97): 20864–20865). We conducted this research prior to the existence of the USDA Forest Service Research and Development Institutional Animal Care and Use Committee (IACUC). Prior to 2015, there was no formal IACUC policy or ethics review. In 2015, United States Department of Agriculture Research and Development adopted the policy to form an IACUC and became an officially registered federal research institution. As a result, this 1991 study is exempt from IACUC because it complied with the 1987 *Guidelines* and 1985 *Principles* cited above and was completed prior to more recent IACUC requirements for approval.

Age one and older wild bull trout (n = 93) were collected in Trestle Creek, Idaho (48° 17’ 33.6” North and 116° 19’ 25.1” West) with a backpack electrofisher using unpulsed direct current. The authorized agency, the Idaho Department of Fish and Game (IDFG), reviewed and approved a Scientific Collecting Permit application authorizing two biologists with >20 years of field experience in collecting and handling fish to collect juvenile bull trout using an approved electrofishing method. Unpulsed direct current was used because it reduces the risk of injury to fish [49]. To reduce fish distress, immediately after capture fish were held in live wells with ambient stream water and fish were not anesthetized. Bull trout were later transferred to a tanker truck with chilled and oxygenated water and transported to UI. At the laboratory, fish were acclimated and held in 500 L troughs with single-pass dechlorinated well water at temperatures less than 10°C. Formalin was added to (50–100 ppm for 1 h) to reduce parasite and fungal infections.

Prior to the trials, fish were initially acclimated to feed with a diet of frozen planktonic crustaceans. Thereafter, fish were fed 2.5–3.0 mm pelleted hatchery feed (BioDiet grower, Longview, WA) twice daily at 2.6% of their body weight. Bull trout were held and acclimated at UI from August 21 until the first trial on September 3 and only feeding fish were used in trials. After trials began in September, fish were fed once each day, typically between 11 a.m. and 3 p.m. Despite the acclimation period, some wild bull trout had difficulty transitioning to the laboratory environment and suffered mortality. Thirteen percent of captured bull trout died (n = 12) so later trials used fewer bull trout than earlier trials. Larger bull trout suffered higher mortality and >50% of mortalities were >120 mm. Lack of concealment shelter reportedly increased metabolic costs of juvenile Atlantic salmon and impaired the performance of larger individuals relative to smaller ones [50]. It is possible a lack of shelter in our aquariums contributed to mortality of larger bull trout. Size of mortalities averaged 109.7 mm TL (range 87–128 mm, SD 19.2 mm) and 11.9 gm (range 6.4–16.7 gm, SD 3.9 gm). To avoid additional stress and handling mortality, we measured and weighed bull trout after the trials only. Surviving bull trout averaged 106.2 mm TL (range 79–143 mm, SD 17.52) and 10.8 gm (range 3–24.5 gm, SD 5.14), five days after the termination of the trials on September 28.

### Wild bull trout trials

Wild bull trout were placed in aquaria and tested serially in multiple trials. Lighting for the trials was provided by fluorescent fixtures regulated on a daily cycle. For each trial, the transition between darkness and daylight included 1 h of dawn during which the light increased linearly from darkness to full daylight. Conversely, the transition between daylight and darkness included 1 h of dusk during which the light dimmed linearly from full daylight to darkness. Full (laboratory) daylight was measured as 2,239 lux, comparable to outdoor daylight on an overcast day. Water temperature for the trials was regulated with supplemental chillers to provide both constant and diurnal cycles. We selected water temperature within the preferred range for juvenile bull trout rearing (4–13°C [5, 7]), with some trials at higher temperatures (13–15°C) (Table 1).

**Table 1:** Experimental design of wild bull trout aquarium trials. Each trial used 16 aquaria.

Test	Date	Fish per tank	Temperature regime °C	Number of substrate sizes	Light level	Number of observations
1	3–5 September	5–6	Fluctuating 4–12	2	12 h light	25
					12 h dark	5
					dawn	3
2	6–8 September	5–6	Fluctuating 4–12	2	24 h light	29
3	9–11 September	5–6	Fluctuating 4–12	2	24 h dark	33
4	12–14 September	4–6	Fluctuating 7–15	2	12 h light	25
					12 h dark	3
					dawn	1
5	15–16 September	3–6	Fluctuating 7–15	2	24 h light	23
6	17–18 September	3–6	Fluctuating 7–15	2	24 h dark	25
7	19–20 September	3–6	Constant 15	2	12 h light	18
					12 h dark	2
					dawn	1
8	21 September	3–6	Constant 15	2	24 h light	10
9	22 September	2–6	Constant 7	2	12 h light	9
					12 h dark	1
					dawn	1
10	23 September	2–6	Constant 7	2	24 h light	10

Number of observations is the total number for the entire trial.

The response variable for all trials was the number of trout observed in one of three activities within the aquaria: concealed in the substrate, resting on the substrate, or swimming (defined as maintaining position in the water column above the substrate, henceforth swimming). During trials, at approximately hourly intervals in the morning (6–7 a.m.) and in the afternoon (3–5 p.m.), an observer carefully inspected each aquarium and recorded the number of fish engaged in each activity. Between 9 and 12 observations were recorded for each aquarium within a 24 h period (Table 1). Identical observations were made in light and darkness by inspecting each aquarium, except in darkness the observer used a flashlight to momentarily spot fish. During both light and dark trials the observer took precautions to avoid being detected by or disturbing fish. On the last day of each trial, we removed, counted, weighed, and measured all bull trout remaining in each aquarium.

The IDFG did not support the return of surviving fish to the wild. As a result, fish were not released after the trials were completed, but were euthanized with a lethal concentration of buffered Tricaine Methanesulfonate (250 mg/L). The carcasses were incinerated at the Department of Agriculture facility on the University of Idaho campus as per their guidance to limiting the transmission of potential pathogens.

We conducted a series of ten, one to three-day trials between September 3 and September 23 (Table 1). Each of sixteen aquaria was filled with an approximately 100 mm deep layer of one of two randomly selected substrates (26–51 or 52–102 mm), resulting in two blocks of eight aquaria with each substrate. We placed between 2 and 6 bull trout in each aquarium and allowed them to acclimate for approximately 12 hours prior to the trials (Table 1).

**Fig 1:**
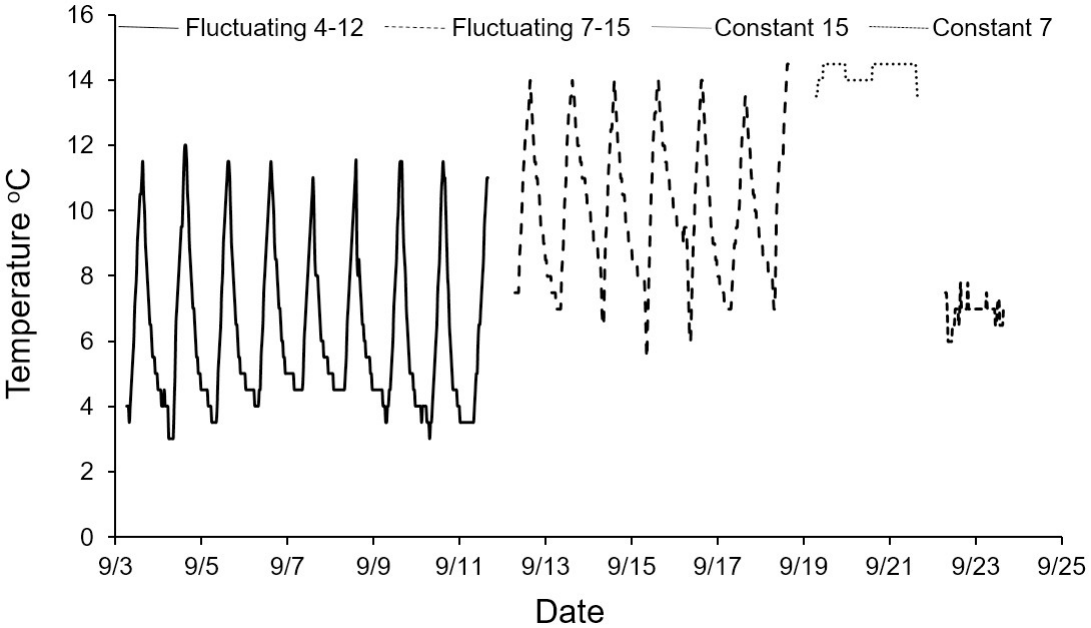
Fluctuating and constant temperature regimes applied during wild bull trout aquarium trials.

Within each trial, we varied the light regime (hours of daylight and darkness) on a 24 h cycle. We exposed fish to 12 h of daylight and 12 h of darkness in four trials, 24 h of daylight in four trials, and 24 h of darkness in two trials (Table 1). The transitions between daylight and darkness also included 1 h of dawn or dusk. We fluctuated water temperature hourly in six trials and held it constant in four trials. Constant water temperature was maintained within +/- 1°C of the target temperature. Water in the fluctuating temperature trials was coldest in the early morning (5–7 a.m.) and increased by about 1°C approximately each hour until peak temperature was attained in late afternoon (4–6 p.m.). Water temperature was then decreased until the minimum was once again attained early the next morning (Fig 1).

We completed 224 individual observations of bull trout in aquarium across three light levels (Table 1). Six observations at dawn, 69 at night, and 149 during daylight. Observations were made by individually inspecting each of 16 aquaria at the assigned time.

### Data analysis

Since individual bull trout activities were recorded as one of three categories, we used a multinomial logistic regression model [51] to examine the effect of the experimental treatments on bull trout behavior. Multinomial logistic regression is similar to binary logistic regression models in that it models the probability of an event yet differs in that it can be used to model multiple categories as a function of predictor variables. Like all logistic regression, the parameters are on a logit scale and require an inverse logit transformation to produce predicted probabilities:

$$\text{Pr}(Y_{i}=K)=\frac{e^{\beta }{}^{{}_{K-1}X_{i}}}{1+\Sigma_{k=1}^{K-1}e^{\beta }{}^{{}_{K}X_{i}}},$$

where *K* is the number of categories, *e* is the exponential function, β is a vector of parameter estimates, and *X* the corresponding predictor variables (*i*). See [51] for more details.

Here we modeled two categorical fish responses, concealed and resting, with swimming as the baseline category. Our experimental design also included repeated observations of multiple fish within individual tanks, hence the observations within a tank were likely to be dependent. To account for tank-level dependence, we fit models with tank-level random effects that were assumed to be normally distributed with a mean of zero [52]. The random effects were nuisance parameters needed to account for within-tank dependence. This dependence is often referred to as a “tank effect” and represents an unknown or unmeasured factor(s) that affected the response (behavior) of fish within a tank. Tank effects are relatively common in behavioral and other experiments that involve housing multiple subjects in the same experimental unit. Random effects are commonly applied to account for the dependence. Failure to account for the dependence would result in biased standard errors, p-values, and measures of model fit. All multinomial logit models were fit using the *mlogit* package [53] implemented in R statistical software version 3.2.2 [54].

To evaluate hypotheses regarding the effect of thermal regime, light regime, and substrate on bull trout behavior, we used likelihood ratio tests (LRT) [51]. Initially we evaluated the support for each treatment effect by fitting a model with all effects, systematically excluding one treatment at a time, and conducting the LRT. Main treatment effects were retained when p< 0.05. We then evaluated all two-way interactions of treatments that were statistically significant using an identical procedure. The model was fit using all two-way interactions, individual two-way interactions were systematically excluded, and the LRT was conducted to determine the statistical significance of the excluded interaction using a critical α level of 0.05. All LRTs were conducted using the *lrtest* function in the *mlogit* package [53]. The final multinomial logit model consisted of statistically significant main treatment effects and two-way interactions. Goodness-of-fit of the final model was assessed using Andrews' [55] omnibus χ2 test, a multinomial version of the Hosmer-Lemeshow test.

The precision of each parameter estimate in the final model was estimated by computing 95% confidence intervals. Parameters with confidence intervals that contained zero were not interpreted because the nature of the relationship could not be determined. To facilitate interpretation, we estimated odds ratios [56] for each predictor variable in the final model with confidence intervals that did not contain zero. The odds ratios were interpreted relative to the baseline category (swimming) used in the model.

## Results

Light regime had the largest influence on wild bull trout concealment behavior. Fish were 14.5 times (1/0.07 Odds Ratio) less likely to be concealed under constant darkness and 4.1 times more likely to be concealed under 12 h light x 12 h darkness regimes, compared to constant light (Table 2). Similarly, fish were 4.1 times (1/0.247) less likely and 4.0 times more likely to be resting on the substrate under the constant dark and 12 h light x 12 h darkness regimes, respectively, relative to constant light. Changes in lighting during the observation period also influenced bull trout behavior. Fish were 3.4 times (1/0.30) less likely to be concealed during dawn, whereas they were 4.4 times more likely to be resting during darkness, compared to constant or 12 h light (Table 2). The final bull trout multinomial logistic regression model with statistically significant treatments and two-way interactions included: temperature regime (χ^2^ = 70.50, 6 df, p< 0.001), light regime (χ^2^ = 53.66, 4 df, p< 0.001), substrate size (χ^2^ = 169.40, 2 df, p< 0.001), lighting during observation (χ^2^ = 169.16, 4 df, p< 0.001), temperature during observation (χ^2^ = 138.39, 2 df, p< 0.001), and the following two-way interactions: light regime by temperature during observation (χ^2^ = 42.00, 4 df, p< 0.001) and substrate size by lighting during observation (χ^2^ = 22.09, 4 df, p< 0.001).

**Table 2:** Parameter estimates, Standard Errors (SE), upper and lower 95% Confidence Limits (CL) and odds ratios from the final multinomial logistic regression model of wild bull trout behavior.

Parameter	Estimate	SE	Lower CL	Upper CL	Odds ratio
Concealment					
Intercept	4.296	0.411	3.490	5.102	
Constant temperature (15°C)	0.672	0.237	0.207	1.137	1.96
Fluctuating temperature (4–12°C)	0.958	0.200	0.567	1.350	2.61
Fluctuating temperature (7–15°C)	0.147	0.155	-0.157	0.451	
Constant dark	-2.676	0.502	-3.661	-1.692	0.07
12 h light/dark	1.419	0.314	0.803	2.034	4.13
Medium substrate (26–51 mm)	-1.825	0.467	-2.741	-0.910	0.16
Dawn during observation	-1.213	0.418	-2.033	-0.394	0.30
Dark during observation	-0.565	0.349	-1.250	0.120	
Temperature during observation	-0.287	0.044	-0.373	-0.201	0.75
12 h light/dark x Temperature during observation	-0.083	0.027	-0.136	-0.031	0.92
Constant dark x Temperature during observation	0.204	0.042	0.122	0.285	1.23
Medium substrate (26–51 mm) x Dawn during observation	0.134	0.544	-0.932	1.200	
Medium substrate (26–51 mm) x Dark during observation	0.577	0.165	0.254	0.899	1.78
Random effect	1.001	0.334			
Resting					
Intercept	1.813	0.533	0.729	2.897	
Constant temperature (15°C)	0.232	0.221	-0.202	0.666	
Fluctuating temperature (4–12°C)	0.388	0.151	0.091	0.685	1.47
Fluctuating temperature (7–15°C)	0.004	0.154	-0.298	0.306	
Constant dark	-1.403	0.380	-2.148	-0.658	0.25
12 h light/dark	1.392	0.309	0.786	1.998	4.02
Medium substrate (26–51 mm)	0.791	0.420	-0.033	1.615	
Dawn during observation	0.262	0.446	-0.611	1.135	
Dark during observation	1.471	0.462	0.566	2.376	4.35
Temperature during observation	-0.154	0.027	-0.206	-0.102	0.86
12 h light/dark x Temperature during observation	-0.087	0.026	-0.139	-0.036	0.92
Constant dark x Temperature during observation	0.103	0.033	0.039	0.167	1.11
Medium substrate (26–51 mm) x Dawn during observation	-0.054	0.529	-1.091	0.983	
Medium substrate (26–51 mm) x Dark during observation	-0.165	0.325	-0.802	0.472	
Random effect	1.539	0.363			

Parameters should be interpreted relative to swimming behavior as the baseline response.

**Fig 2:**
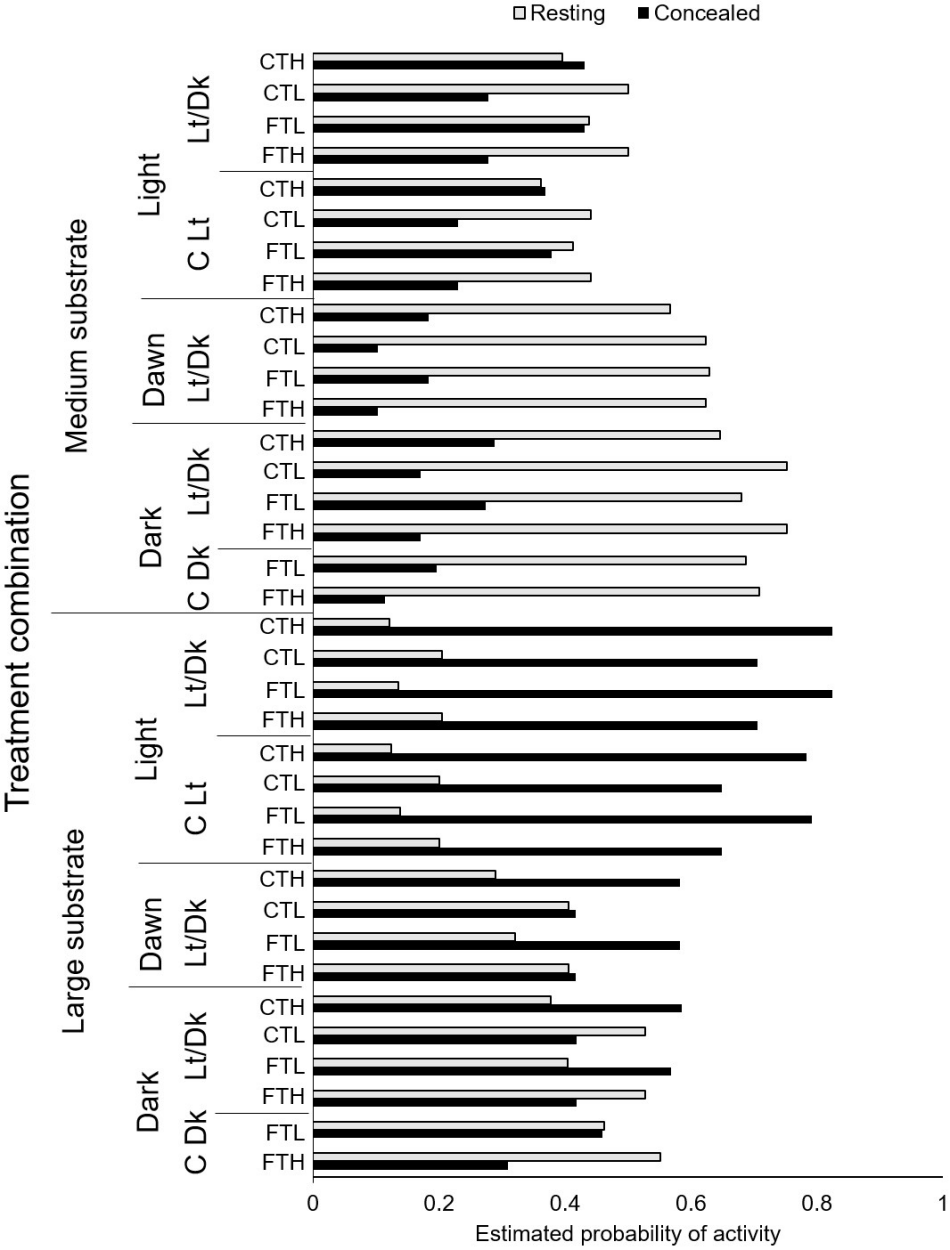
Estimated probability of concealed and resting behavior of wild bull trout for each treatment combination of substrate size, light during observation, light treatment (constant dark, C Dk; constant light, C Lt; 12 h light and dark, Lt/Dk), and temperature regime (constant at 15C, CTH, constant at 7 C, CTL, fluctuating at 7-15C, FTH; fluctuating at 4-12C, FTL).

A majority of wild, juvenile bull trout concealed in substrate during daylight. When fish had access to large substrate during daylight, from 70 to 82% concealed (Fig 2). The largest proportions of bull trout concealed during fluctuating low temperatures and constant high temperatures (Fig 2). In contrast to daytime observations, in darkness from 46 to 55% bull trout rested on top of large substrate (Fig 2).

Substrate size as well as light influenced bull trout behavior. The parameter estimates for concealment behavior suggest that fish were 6.2 (1/0.16) times less likely to be concealed in tanks containing medium (26-51mm) substrate compared to larger (52–102 mm) substrate. However, light level remained important, the interaction between medium size substrate and light regime suggested fish were 1.8 times more likely to be concealed in medium sized substrate during darkness (Table 2). During daylight from 23 to 38% of bull trout concealed compared to at night when from 69 to 71% of bull trout rested on medium substrate (Fig 2).

**Fig 3:**
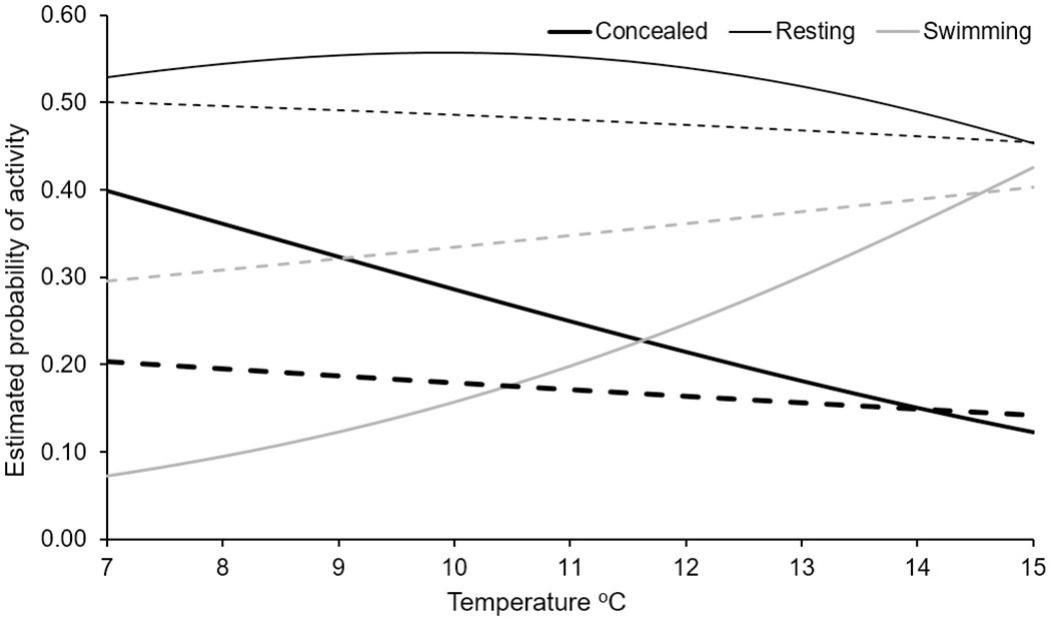
Estimated probability of concealed, resting, and swimming behavior of wild bull trout for large substrate and fluctuating temperatures in the ranges shown. Wild fish predictions assumed behavior occurred during dark for the 12 h light and 12 h dark (solid lines) and constant dark (dashed line) treatments.

Water temperature, in combination with light regime, had a strong influence on bull trout behavior. On average, with increasing temperatures, both concealment in- and resting on- substrate decreased and swimming increased (Figs 2 and 3). However, water temperature during observation had a larger effect on fish that experienced 12 h light x 12 h darkness regimes as compared to fish exposed to constant darkness (Figs 2 and 3). Although thermal regime was statistically significant, it had a much smaller effect than the other factors (light level and substrate) we evaluated (Fig 2). As water temperature increased from 7 to 15°C, bull trout were less likely to conceal in substrate, the probability of swimming increased, and the probability of resting declined slightly (Fig 2).

Random-effects terms were significant (based on parameter estimates and SE) in most models (Table 2). These results suggest there was tank level dependence or a “tank effect”. As described above in Data analysis, we used random effects terms as nuisance parameters to address tank dependence.

## Discussion

Movement of wild salmonids into concealment cover is well documented and may be influenced by a variety of factors including water temperature, light, available cover, predators, or food availability. Our results confirm that water temperature, light, and substrate have a substantial effect on bull trout concealment under experimental conditions.

As water temperatures decline, salmonids often migrate from summer habitat into other portions of watersheds [57] where they typically select areas of low water velocity and enter concealment cover [6, 10, 11, 35, 36, 39, 58]. Temperature thresholds differ among species and influence periods of activity and hiding [35, 39, 40]. The water temperature regimes we examined (4–13°C) were predominately within the preferred ranges for juvenile bull trout rearing, with some trials at higher temperatures (13–15°C). Bull trout are among the most thermally sensitive species residing in coldwater western North American streams [7]. Laboratory studies [59] corroborate field investigations suggesting bull trout have among the lowest upper thermal limits and growth optima of North American salmonids. The ultimate upper incipient lethal temperature (UUILT) for bull trout was similar to that of Arctic Char (*Salvelinus alpinus*) and 1–5°C lower than those reported for brook, rainbow, and brown trout and Pacific salmon [59]. The probability of juvenile bull trout occurrence declined at higher temperatures and fish were most likely to occur in summer mean temperatures of 6–9°C, summer maximum 7 day means of 8–10°C, or single summer maximums of 11–14°C [5]. The duration of high temperatures was also important, juvenile bull trout were more likely to occur in sites where the daily mean exceeded 12°C for less than 15–30 days [5]. Others have similarly reported that juvenile bull trout were rarely found where mean summer temperatures exceed 12°C [7, 60]. Reported thresholds for bull trout (3°C; [6]) were lower than the lowest constant temperature we evaluated (7°C). Nonetheless, at temperatures ranging from 7–9°C, we observed that on average, 30–40% of bull trout concealed under a 12 h light/12 h dark regime. Peak bull trout growth was reported at 13.3°C [59] and at this temperature, less than 20% of bull trout concealed in substrate within our aquaria. Consequently, our observations suggest that temperatures above 3°C may influence fish detection, especially during underwater snorkel counts [61, 62].

Light also has a strong influence on the concealment behavior of salmonids. Goetz [63] reported that juvenile bull trout were primarily nocturnal and night snorkel counts exceeded day counts 2–4 fold, suggesting bull trout were further from cover and more visible at night. Similarly, juvenile Arctic Char were primarily nocturnal and remained hidden during the day [64] and Stenzel (1987- cited in [64]). Similarly, we found that the largest proportion of bull trout concealed under simulated daylight for 12 h light/12 h dark, whereas the least amount of concealment occurred under 24h dark conditions. Previous studies hypothesized that such diurnal changes in juvenile salmonid concealment behavior may provide a means to avoid predation [11, 65–67]. For example, trout fry drastically changed habitat preferences in the presence of predators by seeking cover and restricting feeding [68]. Such diurnal changes in behavior can also affect the ability to detect fishes and results in systematic underestimates of fish abundance.

Movement of bull trout into concealment cover may also be influenced by available cover. Juvenile bull trout are typically cryptic and associated with concealment cover consisting of gravel-rubble substrate and organic debris [44, 69, 70]. Juvenile salmonids may shift substrate preference as water temperatures decrease and photoperiods shorten [40, 57]. We similarly found that juvenile bull trout were more likely to conceal when the substrate was larger, and the effect of substrate size changed with photoperiod. Because our fish were exposed to a single gravel size in each treatment, we cannot interpret the relationship between substrate size and concealment as a preference. Rather, we believe that substrate size reflected the ease at which fish could burrow into the substrate. Larger substrates had larger interstitial spaces as compared to smaller substrates which enhanced fishes’ ability to burrow into larger substrates. Thus, we expect that more fish to conceal in streams containing substrate with larger interstices spaces.

Postulated reasons for concealment behavior include protection from floods or ice [71], reduced energy expenditure [38, 43, 48, 71], and avoidance of predators [72] or cannibalism [41]. Conversely, although bull trout may emerge from concealment at night [10, 13] the mechanisms for this diel behavioral emergence are also poorly understood. Winter salmonid behavior has also been described as a “cost-minimizing shelter-and-move strategy” [43]. Daytime winter concealment and nocturnal activity of brown trout reduced the risk of habitat exclusion and microhabitat inclusion by ice formation while daytime sheltering reduced energy expenditures and the risk of predation, suggesting an ecologically adaptive homeostatic behavioral response to buffer the energy deficit and adverse environmental conditions in winter [43]. Power [73] described the glacial conditions bull trout evolved in and suggested nighttime foraging may have developed in response to fluctuating water clarity. Warmer daytime temperatures increase glacial meltwater and erosion of glacial sediments resulting in high transported sediment levels that reduce water clarity [73] in contrast, colder nighttime temperatures may result in less sediment transport and higher water clarity. Unfortunately, we did not feed bull trout at night so were unable to assess the effects of food availability on nighttime bull trout emergence from concealment.

Several fruitful areas of research remain to be explored in order to more fully understand the mechanisms of daytime concealment and nighttime emergence by bull trout and other salmonids. For example, although logistically challenging, controlled experiments might be applied to examine the influence of predators and food availability on salmonid diel activity.

### Implications for detection

Concealment of bull trout influenced the detection efficiency of both mark-recapture and depletion electrofishing estimates [31, 74] and concealment may also limit the species detection during daytime snorkel surveys. Bull trout daytime snorkeling efficiency averaged 12.5% and snorkel detection rates equaled 6%, 15%, and 27% for bull trout from 60–99 mm, 100–199 mm, and 200–350 mm total length, respectively [34]. Our aquarium trial results confirmed that when fish had access to large substrate, most juvenile bull trout concealed during daylight. In contrast to daytime observations, in darkness most bull trout emerged and were resting on the substrate or swimming in the water column. Bull trout sampling protocols may be improved by applying concealment observations to conduct surveys when bull trout are most likely to be resting on the substrate or swimming in the water column. When substrates are large enough to provide sufficient-sized interstitial areas, many juvenile bull trout will conceal during daylight. The proportion of juvenile bull trout visible on the substrate or in the water column decreased from darkness to dawn to daylight. Consequently, a larger proportion of juvenile bull trout will be detectable if nighttime snorkel counts are conducted. Even at night, however, a portion of the population may remain concealed if large enough substrate is present. As a result, it is essential to adjust nighttime snorkel counts with empirically derived detection rates or by modeling snorkeling efficiency [31, 34]. When nighttime snorkel surveys are considered too dangerous or impractical, daytime detection rates may be improved by 1) completing snorkel surveys when water temperatures exceed 11°C, and 2) adjusting daytime snorkel counts with empirically derived detection rates or modeling efficiencies.

### Movements into the water column

We were surprised to observe more bull trout swimming in the water column as daytime temperatures increased toward 15°C. Entering the water column during the day is unusual behavior by fish that typically exhibit cryptic behavior [8, 10]. As water temperatures increased, it is possible juvenile fish became thermally stressed above a preferred temperature range or experienced increased metabolic needs. The fundamental thermal niche for bull trout is from 10.2–14.3°C compared to their maximum-growth temperature range of 10.9–15.5°C [59]. Temperatures near the growth optimum are considered close to the upper limits for bull trout occurrence [59]. Field observations have confirmed declines in bull trout abundance in northern Idaho streams when maximum summer water temperatures exceeded 14°C [75]. Metabolic rate, however, tends to increase exponentially with increasing temperature [76], and at higher temperatures bull trout may have actively sought more food to meet increasing metabolic needs.

Movements into the water column at higher temperatures may also have been influenced by fluctuating temperatures. Several studies illustrate that the magnitude of daily temperature variations can affect salmonid stress levels [77]. However, the mechanisms underlying the response of fish to temperature fluctuations are unclear and probably result from: the stress associated with life in cycling thermal regimes, costs associated with increased exposure to temperatures near an upper lethal limit, or an interaction between the two [78]. A growing number of studies underscore the importance of temperature variability to salmonid ecology [77].

### Study limitations

Our results have some important limitations. We were unable to assess bull trout response across each of six possible light levels (dusk, darkness, midnight, dawn, light, and mid-day) because observations were imbalanced between light, darkness, and dusk and dawn transitional periods between light and darkness. Since fluctuating temperature trials were completed initially and constant temperatures trials subsequently, we were also unable to compare fish responses to the identical levels of each variable (temperature, substrate, and light). In darkness, we assumed fish remained in their preferred location and some fish may have moved in response to the observer. Small aquaria prevented us from offering multiple substrates, other researchers (e.g., [64, 79] reported that fish selected preferred substrates when given a choice. We used the same bull trout serially in multiple trials and mortality of fish resulted in fewer fish in later vs earlier trials.

Bull trout concealment behavior was evaluated under a somewhat limited range of water temperatures, light conditions, and substrate sizes, and our controlled laboratory results may not be directly applicable to natural conditions. We encourage more rigorous testing of the influence of these three environmental variables, as well as other factors (i.e., predation and cannibalism), on bull trout concealment. Other biologists have confirmed the importance of studying wild fish behavior or, at a minimum, studying fish raised in more complex habitats [e.g., 80]. Despite the limitations of our study, results confirm that water temperature and light have a substantial effect on bull trout concealment. This information may be applied to improve field sampling protocols, increase detection efficiencies, and enhance knowledge of bull trout niche requirements.

## Supporting information

S1 Data Metadata for wild bull trout data for analysis. (CSV)

S2 Data Wild bull trout data for analysis. (CSV)

S1 Checklist NC3Rs ARRIVE guidelines checklist. (DOCX)
