# Supplementary material for: Concealment of juvenile bull trout in response to temperature, light, and substrate: Implications for detection
Source: PLoS One. 2020 Sep 4;15(9):e0237716. doi: 10.1371/journal.pone.0237716 (PMC7473556; doi:10.1371/journal.pone.0237716)

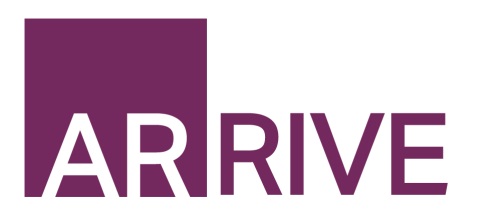


The ARRIVE Guidelines Checklist

Animal Research: Reporting In Vivo Experiments

Carol Kilkenny^1^, William J Browne^2^, Innes C Cuthill^3^, Michael Emerson^4^ and Douglas G Altman^5^

*^1^The National Centre for the Replacement, Refinement and Reduction of Animals in Research, London, UK, ^2^School of Veterinary Science, University of Bristol, Bristol, UK, ^3^School of Biological Sciences, University of Bristol, Bristol, UK, ^4^National Heart and Lung Institute, Imperial College London, UK, ^5^Centre for Statistics in Medicine, University of Oxford, Oxford, UK.*

|  | | ITEM | RECOMMENDATION | Section/ Paragraph |
| --- | --- | --- | --- | --- |
| 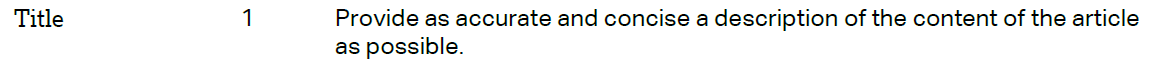 | | | Title |  |
| 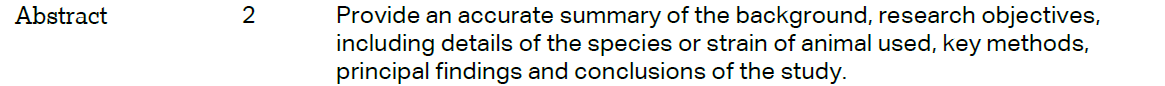 | | | Abstract |  |
| INTRODUCTION | | |  |  |
| 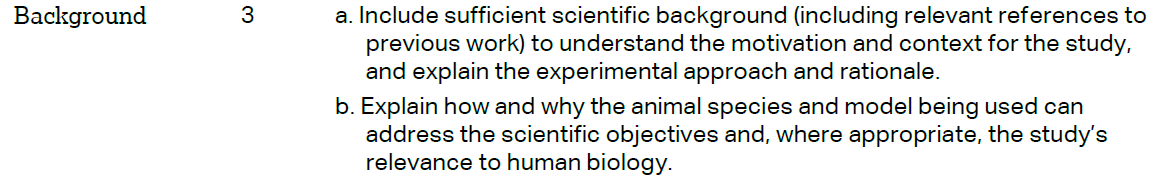 | | | Paragraphs 1-3  Paragraphs  4-6 |  |
| 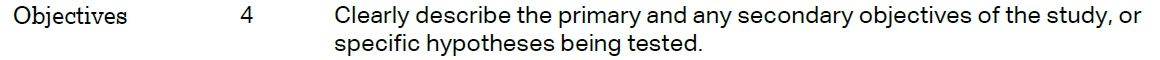 | | | Paragraph 6 |  |
| METHODS | | |  |  |
| 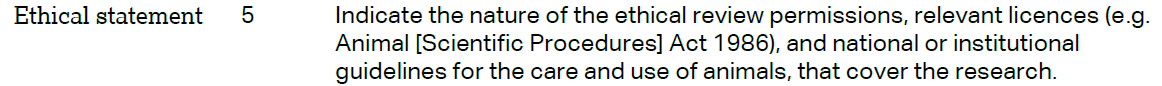 | | | Fish source and care: Paragraphs 1,2 |  |
| 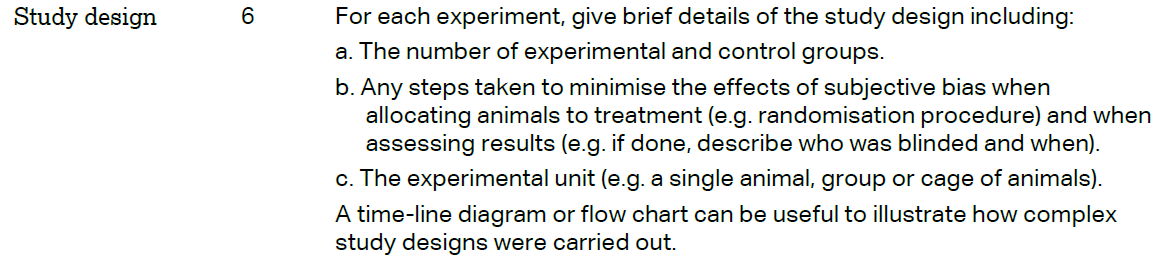 | | | Paragraph 1  Fish source and care:  Paragraphs 1,2  Paragraphs  1,3-4 |  |
| 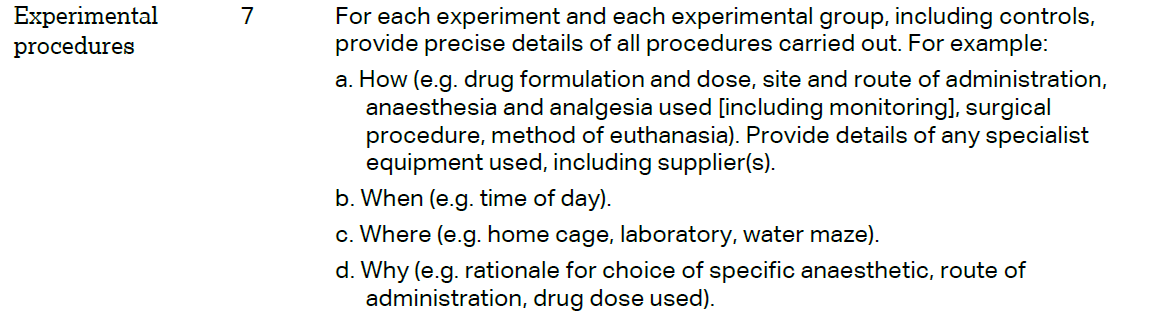 | | | Paragraphs  5-10  Wild bull trout trials:  Paragraphs 1-6 |  |
| 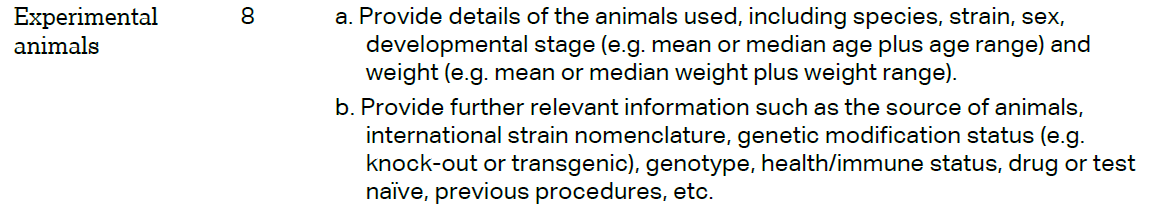 | | | Fish Source and care:  Paragraphs 2,3 |  |

The ARRIVE guidelines. Originally published in *PLoS Biology*, June 2010^1^

| 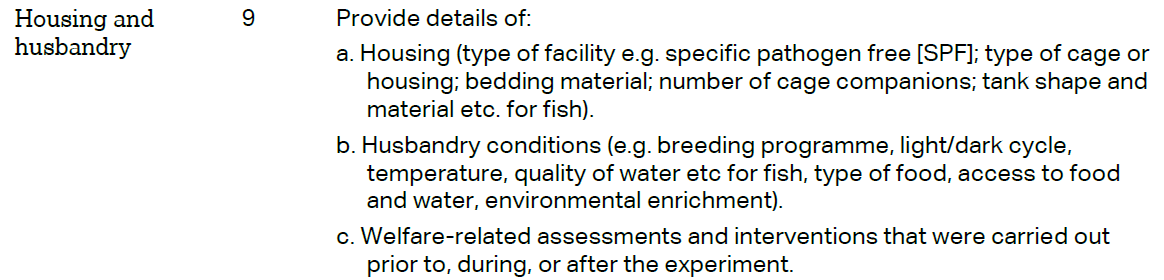 | Paragraph 1  Fish source and care:  Paragraphs 1-3 | |
| --- | --- | --- |
| 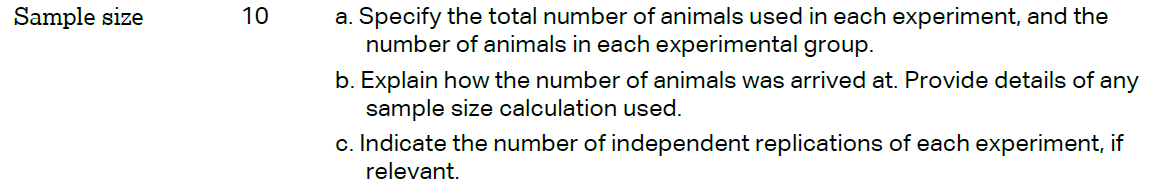 | Wild bull trout trials:  Paragraphs 4-6 | |
| 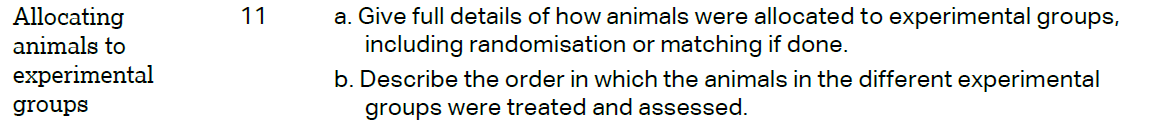 | Wild bull trout trials:  Paragraphs 1-6 | |
| 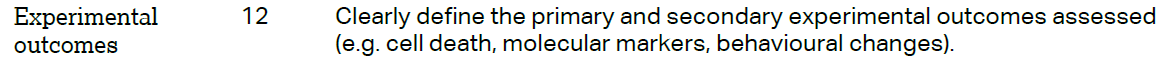 | Wild bull trout trials:  Paragraphs 1,2 | |
| 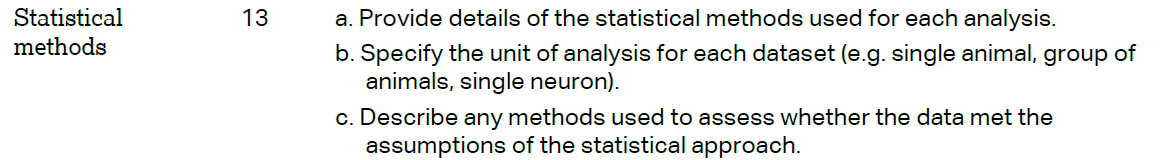 | Data analysis:  Paragraphs  1-3 | |
| RESULTS |  | |
| 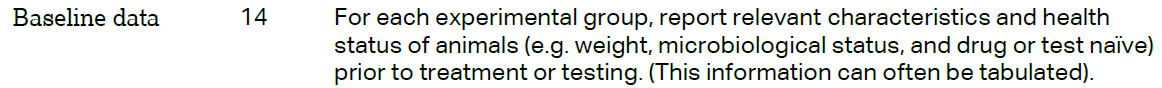 | Fish source and care:  Paragraph 3 | |
| 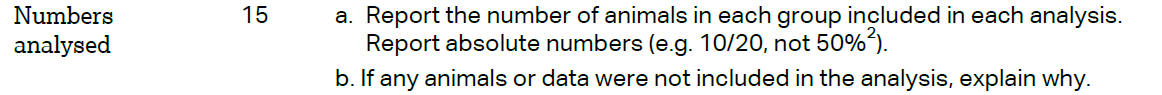 | Wild bull trout trials:  Paragraphs 4-6 | |
| 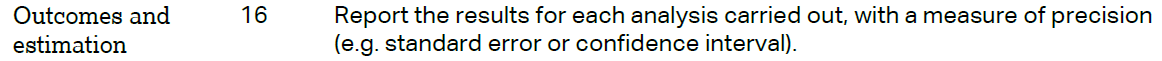 | Paragraphs 1-5 | |
| 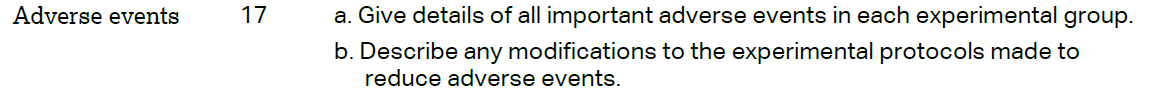 | Paragraph 5 | |
| DISCUSSION |  | |
| 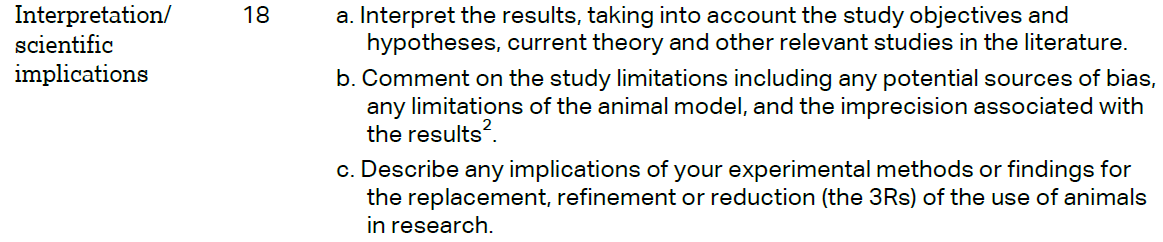 | Throughout  Study limitations: Paragraphs 1,2  Paragraphs 5,6 | |
| 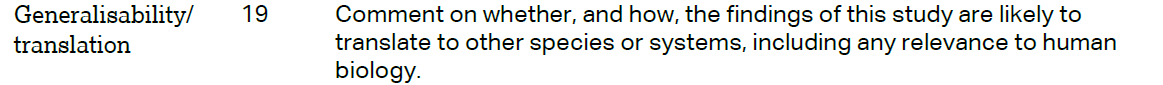 | Paragraphs 2,6 | |
| 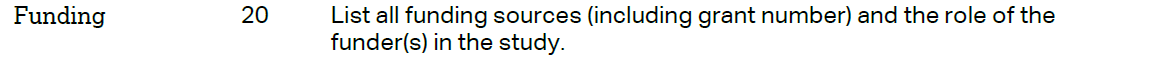 | | Acknowledgements: Paragraph 1 |


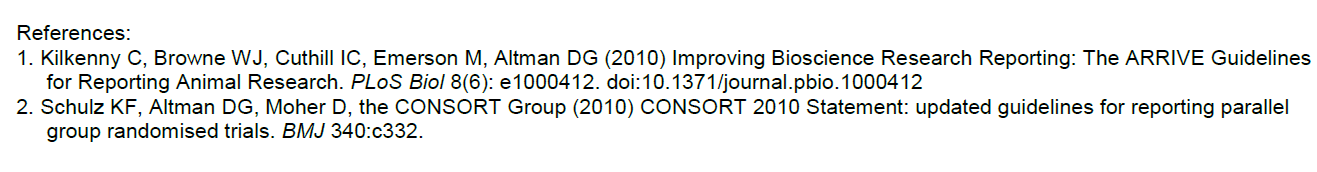

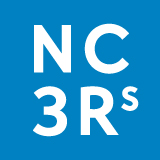

Supplement: S1 Checklist — (DOCX) [file pone.0237716.s003.docx]
